# Interconnected Pathways: Postural Stability and Vocabulary Skills in Preschool-Aged Children

**DOI:** 10.3390/children10121891

**Published:** 2023-12-06

**Authors:** Giulia Calignano, Giorgia Lettere, Irene Leo, Francesca Maritan, Laura Mattera, Patrizia Granata, Daniela Lucangeli, Eloisa Valenza

**Affiliations:** 1 Department of Developmental and Social Psychology (DPSS), University of Padova, 35131 Padua, Italy; giorgialettere@libero.it (G.L.); irene.leo@unipd.it (I.L.); daniela.lucangeli@unipd.it (D.L.); eloisa.valenza@unipd.it (E.V.); 2 “Giovanni XXIII” Centro Infanzia, 35142 Padua, Italy; francesca.maritan@pecpsyveneto.it (F.M.); laura.mattera@pecpsyveneto.it (L.M.); coordinatrice@scuolainfanziamandria.it (P.G.)

**Keywords:** motor development, language acquisition, network analysis

## Abstract

Previous research has highlighted an interplay between postural abilities and linguistic skills during infancy. However, this relationship could undergo further radical transformations in other periods of development. This current study explored a plausible network of relationships among postural abilities and vocabulary skills in a substantial cohort (N = 222) of preschoolers aged between 2 and 5 years—a developmental phase critical for refining both language and motor competencies. Here, postural stability was measured in terms of balance duration and accuracy, alongside an assessment of comprehension and expressive vocabulary skills. Employing a diverse set of techniques, i.e., data and missing data visualization and multilevel regression analysis, task complexity and age emerged as crucial factors explaining our data. In addition, network analysis indicates that language production plays a central role within postural and language interdomain networks. The resulting discussion focuses on the useful implications of this study for the assessment of typical preschool development, which would benefit from tailored methodological inspections guided by developmental theories that are framed in inter-domain approaches.

## 1. Introduction

Several studies previously investigated the link between language acquisition and sensorimotor experiences [1,2,3]. Indeed, language acquisition is intricately tied to perceptual, computational, social, and neural mechanisms [4], extending into whole-body movements [5,6,7,8]. Early motor milestones, such as sitting and independent walking, provide pivotal opportunities for interaction, learning, and social engagement, shaping language acquisition [7,9,10]. Simultaneously, exposure to language from birth, alongside motor experiences, influences the timing and content of language acquisition, accentuating critical connections between objects, actions, and thoughts [11]. Beyond infancy, toddlers and children engage in increasingly richer conversations [12], where emerging motor skills prompt caregivers to introduce and reinforce the comprehension of new actions, thereby fostering a reciprocal relationship [13]. 

By focusing on preschool age and early childhood, the intricate network connecting language and motor domains seems to undergo critical shifts before and after the third year of life [14,15,16]. Previous results have suggested that, in preschoolers aged 18 months to 3 years, early motor performance is strictly associated with subsequent language abilities, whereas early language performance has shown to have a limited predictive role for later motor skills. Conversely, from 3 to 5 years of age, variations in language performance emerge as reliable markers for subsequent motor skills, while differences in motor performance are less likely to predict language skills [17]. In addition, studies on at-risk populations for atypical development, for instance on individuals with a diagnosis of Specific Language Impairment (SLI), reported a delayed attainment of motor milestones, both in gross and fine motor movements, and difficulties in higher-level motor planning, also involving postural skills [18,19,20,21]. A substantial portion of preschoolers diagnosed with a variety of language disorders also manifest motor difficulties, indicating that early language challenges are involved in later fine and gross motor skill development [22]. Likewise, it is widely acknowledged that children with Developmental Coordination Disorder (DCD) may experience speech difficulties [23]. Moreover, when comparing language profiles of children diagnosed with either DCD or SLI to typically developing controls, both clinical groups exhibit distinguishable language profiles [18]. Similarly, in comparing motor profiles of children diagnosed with either SLI or DCD to controls, both clinical groups tend to show substantial difficulties [24,25]. 

### This Study

This study explored the intricate relationship between postural and language skills within a large sample of typically developing children aged between 2 and 5 years, specifically from 29 to 71 months of age. This age range is characterized by notable advancements in both motor and linguistic domains. Around the age of two, humans experience a rapid increase in several pivotal motor and linguistic milestones, thereby enhancing their skill set. At the same time, the preschool period is marked by escalating motor and linguistic demands. Preschoolers increasingly engage in peer interactions, and these interactions, while revealing their lack of proficiency, play a critical role in refining children’s linguistic abilities in terms of comprehension and production [26]. Specifically, the grounded approach of this study regarding language assessment in preschool-aged children focuses on the lexical level as a core ability that allows children to refer and manipulate a number of real entities at a representational level. The present language assessment involves a dual-phase process, conducted in an environment that promotes focus and minimizes distractions. This approach is typified by a sequential evaluation of both receptive and expressive language skills. In the first phase, focusing on receptive language skills, children are individually assessed through tasks that require them to correlate spoken words, such as nouns and verbs, with corresponding images. This phase evaluates the child’s ability to understand and process spoken language. The second phase shifts to expressive language skills, where children are tasked with naming images presented to them. The assessment is designed to adapt to the child’s responses. Immediate feedback is provided: if a child responds incorrectly, the experimenter offers graduated levels of assistance, starting with semantic help and progressing to phonological cues if necessary. This adaptive feedback mechanism not only increases informativity about the assessment’s accuracy but also provides a deeper insight into the child’s specific linguistic strengths and challenges. 

Moreover, this developmental window represents a sensitive period for the enhancement of fundamental postural and motor skills [27], as well as the maturation of sensory and musculoskeletal systems crucial for maintaining postural stability [28].

In particular, the preschool age is characterized by an increase in the postural demands necessary for the execution and control of complex motor activities such as jumping or standing on one foot [29]. Consequently, efficiency in postural stability becomes an informative indicator of the performance of complex motor activities exhibited during this age. This study specifically focused on postural stability, which enables individuals to sustain an intended postural control of their body [30]. Postural abilities necessitate the integration of multiple physiological systems, including the central nervous, visual, vestibular, and proprioceptive systems [31]. In order to further understand the relationships between postural stability, lexical comprehension, and production measurements in preschoolers, this study adopted a network analysis approach, which comprises a set of techniques useful for representing multiple variables and studying their relationships. The basic assumption is that studying the relationships between a plausible number of variables offers better explanations for a complex phenomenon such as the association between postural stability and language performance in preschoolers. This is particularly true when comparing the network analysis approach to traditional correlation studies, which usually limit the investigation to two variables per time. In fact, network analysis organizes the entire data collection into a data matrix and allows for a representation of all the variables of interest as nodes and their relationships as lines (edges). In this way, the concept of the network becomes an analytical and operational tool that uses mathematical language and graph theory to help us define plausible relationships between postural stability and language skills. 

As indicated by several works across a range of psychological domains [32,33,34,35], this study specifies theoretical and empirical plausible relationships assuming a bidirectional association between (a) the lexical level in terms of production and comprehension and (b) efficiency in postural stability measured as (i) the length of time a child was able to maintain their posture (i.e., balance duration) and (ii) the amount of movement that is observed when a child attempts to maintain a static posture (i.e., the number of times the child drifted or wavered while standing [36]). Participants performed two novel tests on postural ability, holding specific poses of increasing difficulty, both with open and closed eyes, during which their posture accuracy and duration were measured using a balance board. This allowed us to conduct analyses investigating the influence of age, task difficulty, and visual information availability on postural stability performance. The effect of age, treated as a continuous variable in months, was expected to be associated with both linguistic and postural performance. Also, an effect of task difficulty on postural performance was expected, given that the ability to maintain postural stability in a certain position decreases as the available support base reduces and when there are variations in the alignment of body segments, such as the arms [37]. Lastly, an effect of visual information availability on stability performance was expected, because previous studies showed that closed eyes reduced postural stability [38] in young adults [39], children [40], and infants [41], since visual cues play a prominent role in the control of static postural equilibrium, particularly in the first 6 years of life [42]. 

Furthermore, in this study, the chosen method of describing and deriving information was that of visually examining the distribution of data and missing data at an observational level for each participant. Visualizing whether missing data are randomly distributed or dependent on external (i.e., the task) or internal (i.e., age) variables allows for a better interpretation of the data collected. This study aimed to offer a description of data regarding relationships between linguistic and postural abilities, taking into account individual and age differences.

## 2. Materials and Methods

To assess postural abilities, the Nintendo™ Wii Balance board (Redmond, WA, USA), a wireless device connected via Bluetooth to a nearby ASUS Transformer Book Flip TP550L (Fremont, CA, USA) was used. A laptop was used to measure static postural stability. Data were recorded using GPA software version 8 (Global Postural Analysis), which served as an interface between the laptop and the balance board. The balance board was equipped with four pressure sensors placed at the four corners, which allowed it to detect every slight postural movement and continuously determine foot position and weight distribution [43]. The Wii Balance board has a good time resolution (60 Hz), recording a data point every 20 ms. Therefore, it is a valid tool for measuring static postural balance. The Wii balance board is also low cost, easily transportable, and can be applied in research and clinical settings [36,44,45,46]. 

To assess linguistic abilities, lexical comprehension and production were evaluated by means of the Phono Lexical Test [47], a standardized test for the qualitative and quantitative analysis of the receptive and expressive vocabulary of children aged 2.6 to 6 years. The test consists of two sub-tests that individually assess lexical comprehension and lexical production. 

Specifically, the lexical comprehension test consists of 45 sets of illustrations. Each set contains four images, including a target stimulus, a phonological distractor, a semantic distractor, and an unrelated distractor. The stimuli are sorted in descending order on the basis of frequency of use. The child is asked to indicate the illustration that represents the word named by the experimenter. 

The receptive and productive vocabulary size were measured in this manner, accounting for the rate of phonological and semantic errors made by participants and the aids given by the experimenters [47].

### 2.1. Participants

The participants (N = 222; 112 females) were Italian-learning children between 29 and 71 months (2–5 years of age) with an average age of 49.93 months (SD = 11.34). All of the children reported no neurological or clinical condition and were recruited from four private schools in Veneto region, Padova, Italy. In our study, the sample consisted of children belonging to the middle to upper socioeconomic class. This classification is based on their families’ economic possibility to pay tuition fees for private education, indicating a certain level of economic stability and resource availability. Before starting data collection, the parents signed informed consent forms. This study was conducted in accordance with the Declaration of Helsinki and approved by the University Ethics.

### 2.2. Procedure

Postural tasks. The children were individually observed in a quiet room in their schools by two blinded experimenters. After becoming familiar with the two experimenters, the participants were evaluated via static postural tasks. An experimenter showed each child the typical poses of two animals, called the bird and the giraffe poses, and then asked him/her to imitate those poses.

The bird pose consisted of four poses with a static leg position (standing on one foot with one leg bent at about 90°), whereas the position of the arms increased in difficulty from the first to the last pose: (1) the arms open and parallel to the floor; (2) the arms extended along the hips; (3) the arms crossed over the chest; (4) the arms in motion, initially open, then along the hips, and finally crossed in the chest.

Similarly, the giraffe pose had four poses with the same leg position (standing on one foot with one leg extended forward), whereas the position of the arms increased in difficulty from the first to the last pose: (1) with the unilateral arm open and parallel to the floor and the contralateral arm up; (2) with the unilateral arm extended at the hips and the contralateral arm up; (3) with both arms crossed over the chest; (4) with the arms in motion repeating the movement of the previous positions.

Importantly, the children were asked to perform each pose in two different modalities: with open and closed eyes. Before beginning data recording, each participant was asked to step on a balance board with both feet and then to step off of it in order to calibrate it so as to be able to reset the sensors. To easily indicate which foot to hold and which to lift, two colored footprints were placed on the balance board (i.e., an orange footprint for the right foot and a yellow footprint for the left foot). When the child understood the task, s/he was invited to stand on the Wii balance board without shoes and to perform each pose. The task required maintaining posture if possible. The time it took the child to maintain their posture on the balance board was recorded by the GPA program. If the examiner noticed signs of fatigue in the child, s/he interrupted the test, giving him/her the opportunity to play in the room. Each pose was presented as play in such a way as to motivate the child to perform the tasks and ensure the best performance. The balance duration for the postural stability task was approximately 25–30 min. The children completed all the postural stability tasks in about 10 min. To analyze postural stability, two indicators were considered: balance duration (BD), as the time spent on the balance board in a specific pose; and balance accuracy (BA), as the set of movements in different directions (drifting and wavering) that the child uses to maintain a static posture. In particular, the GPA software version 8 recorded the number of “drift”, as the inclinations of the body related to the time trying to maintain the correct posture, and the number of “waver”, as the oscillations of the body resulting from postural adjustments during position maintenance [30]. Then, the GPA calculated the “Power Index”, an accuracy index that determines the numbers of body fluctuations (front–back, right–left) made while assuming the required pose on the balance board. The higher the value, the lower the balance accuracy was.

Language tasks. The TFL test was administered in a quiet and bright classroom in the children’s school. Each child was tested individually. The tasks were presented in the following order: lexical comprehension subtest first and lexical production second. For each subtest, the experimenter presented two example items in the booklet provided by the manual. As soon as the child showed that s/he understood the tasks, the experiment began. The test session lasted about 30 min. During the lexical comprehension task, the experimenter presented the booklet with a set of images to the child, asking him/her to indicate the image corresponding to the noun/verb pronounced by the experimenter. When the answer was wrong, the experimenter corrected him/her, pointing to the correct image. During the lexical production task, the experimenter presented the booklet with the set of images to the child, asking him/her to name the indicated image. If the child did not answer correctly, the experimenter first provided him/her with semantic help, and if s/he made a new mistake or did not answer, the experimenter gave him/her phonological help provided for that item on the registration protocol. Finally, if the correct answer was not given, the experimenter named the image and moved on to the next element.

To measure the comprehension scores with the TFL registration protocol, the number of figures correctly indicated by the child were registered and scored as one point. The raw total score was obtained (with a maximum score of 45). To measure the production scores, the child’s answers were reported on the scoring sheet in the following ways:

- A (+) is marked in the box “1, answer given without help” when the child answers correctly without help.
- A (−) is marked in the box “1, answer given without help” when the child does not answer. If, on the other hand, he gives an unexpected answer, write the answer produced in the space “answer provided”.
- A (+) is marked in box “2, answer provided with semantic help” if the child responds correctly to the semantic help given by the examiner.
- A (+) is marked in box “3, answer provided with phonological help” if the child responds correctly to the phonological help given by the examiner. Each (+) sign is worth 1 point; each (−) sign is worth 0 points. By summing the answers, it is possible to obtain a raw total score of the correct answers without help, with semantic help, and with phonological help.

### 2.3. Statistical Analysis

Data were analyzed by taking advantage of three different statistical approaches by using R free Software version 4.2.1 [48]. First, this study extensively inspected data and missing data as a privileged source of information as a robustness check of our design and the related data collection. To do so, the “ggplot” package [49] and the “naniar” package [50] were used.

Second, Generalized Linear mixed-effects (GLMs) models were implemented in order to estimate the impact of task manipulations (visual modality, postural pose difficulty, and age) on balance duration and balance accuracy. In the postural and lexical data analysis, individual differences were specified, and age was treated as a continuous predictor. This study purposefully considered GLMMs to account for both random (i.e., participants, poses) and fixed effects (postural pose difficulty, visual modality, and age) and to specify the residual distribution family. When needed, this allows one to overcome the assumptions made by ANOVA and LMs that are usually violated when dealing with nonnegative behavioral data (e.g., balance duration and accuracy). Thus, the distribution family was chosen based on residual distributions. All of the models were fitted with the lme4 package [51]. To find the best approximation of the true model, a stepwise forward model comparison approach [52] was followed, which was based on AIC (Akaike Information Criterion) and especially delta AIC and AIC weight as indexes of goodness of fit. The AIC and the AIC weight give information on the relative evidence (i.e., likelihood and parsimony) so that the model with the lowest AIC and the highest AIC weight should be preferred [53]. The model comparison included the simplest model and proceeded by adding predictors. That is, for each dependent variable, i.e., balance duration and accuracy, two different models were tested (see the OSF repository).

Lastly, to jointly examine the weighted associations among variables and to appreciate the complex matrix of associations expected in our data collection, network analysis was used as a tool to explore the weighted multivariate correlation across postural and linguistic measures, by dealing with the complexity of distributed networks. The “bootnet” package [54] was used to estimate a model based on multiple subjects. To explore the relationship between the observed factors, a between-subjects network analysis of partial correlations was used among the variable balance duration (duration), balance accuracy in terms of number of shifts (accuracy), the TFL comprehension (C), and production (P) scores. Of note, the first fundamental step to interpreting the results of a network analysis is the centrality of the nodes. That is, if a node has many and strong associations with other nodes, then it is more central within the network compared to other nodes. For each node, the strength and the Expected Influence (EI) [55] were estimated. Three different estimated networks (i.e., standard EBISglasso, a more conservative EBICglasso with a threshold, and unregularized estimation) were compared. This was decided, given that the typical GLASSO algorithm sometimes fails to retrieve the correct model and gives rise to small false-positive edges, which in turn affects the replicability of small edges over different empirical samples [56].

## 3. Results

### 3.1. Descriptive Statistics

**Figure 1:**
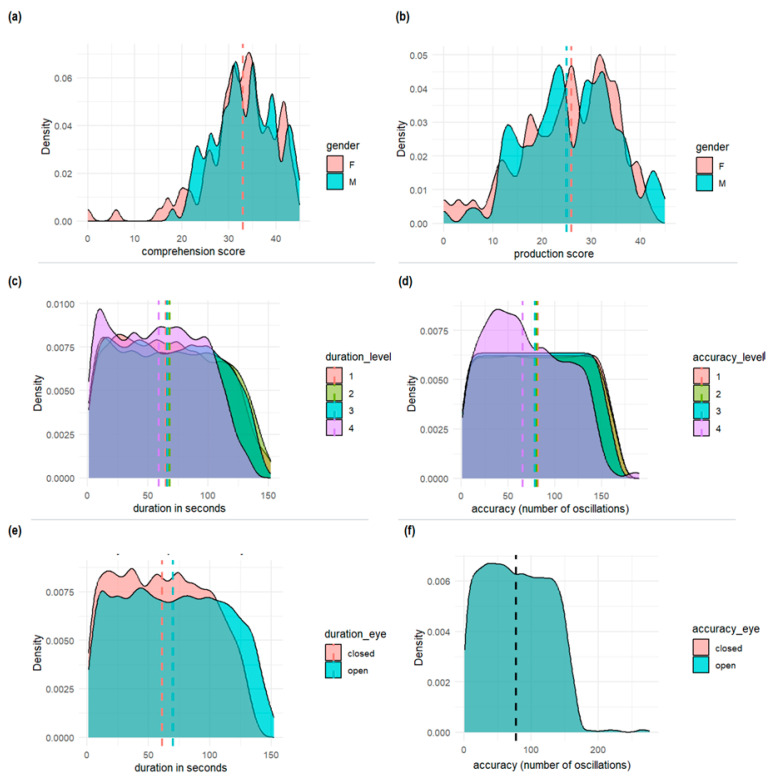
Density plots of (**a**) lexical comprehension scores and (**b**) lexical production scores; balance duration in seconds (**c**) by task difficulty and (**d**) by visual modality (open and closed eyes); balance accuracy (number of oscillations) (**e**) by task difficulty and (**f**) by visual modality (open and closed eyes). For all the plots, the vertical dotted line signals the median value for each group.

Figure 1 shows density plots, which visually display how the present inter-domain dataset is distributed. These plots clearly show where lexical and postural measures are clustered, offering a clear view of the data’s overall structure, central tendencies, and variability, segmented by gender and task manipulation. It is possible to notice that lexical comprehension and production scores were balanced between genders. Moreover, the higher the level of task difficulty (from 1 to 4), the higher the reduction in the duration and accuracy (number of shifts) of the postural balance. Instead, the lack of visual information (closed eyes) only affected the duration of the postural task.

### 3.2. Missing Data Inspection

**Figure 2:**
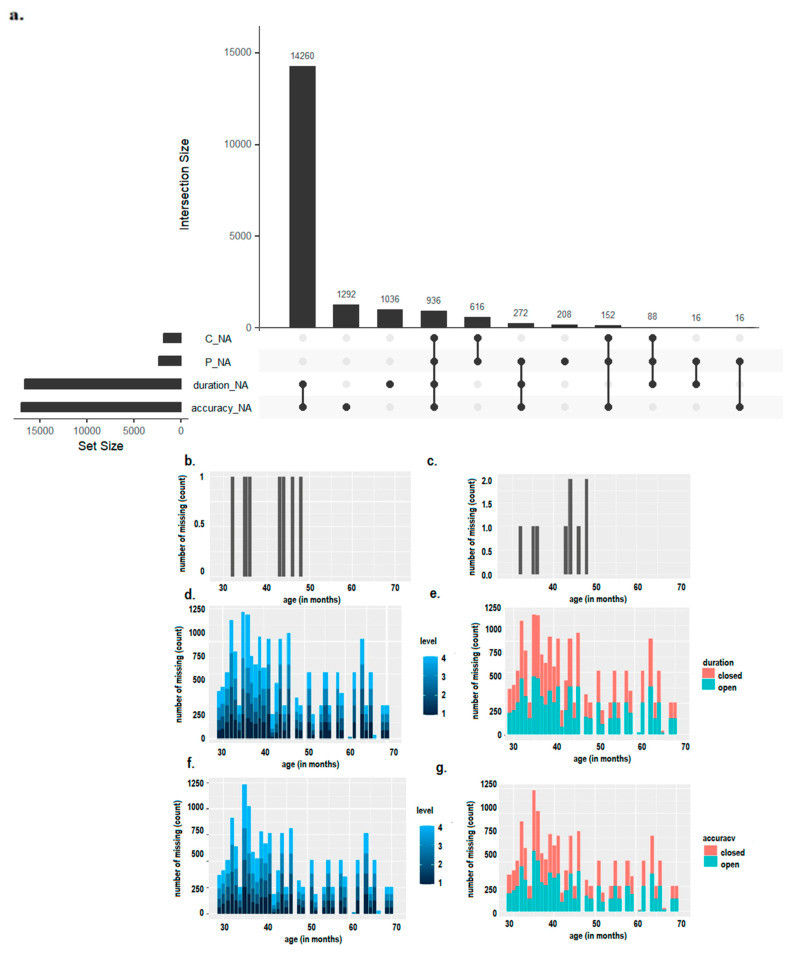
The number of missing values for (**a**) the dependent variable sets: C_NA = comprehension, P_NA = production, duration_NA = balance duration, accuracy_NA = accuracy (number of shift). The number of missing data are split for (**b**) Production, (**c**) Comprehension, (**d**,**e**) Balance duration, and (**f**,**g**) accuracy (number of shifts) across difficulty levels (from 1 to 4) and visual information availability (open and closed eyes).

Figure 2a illustrates the distribution of missing data for each dependent variable, which is the amount of unreported data in each trial from both of the assessments of lexical comprehension and production, as well as postural duration and accuracy.

Within the language domain, our visualization suggested that children displayed a greater amount of missing data during language production tasks in Figure 2b, compared to language comprehension tasks in Figure 2c. This pattern was pronounced in toddlers below 50 months of age (approximately 4 years). Intriguingly, while missing data were predominantly observed in younger children (<50 months) during lexical assessments, as shown in Figure 2b,c, those during the postural tasks were similarly distributed across various age groups as shown in Figure 2b–g. Also, within the postural assessment, the frequency of missing data increased with task complexity, ranging from tasks 1 to 4 shown in Figure 2d–f (the lighter color indicates the greater complexity of the postural task), and without visual information accessibility, signifying the closed-eye tasks, which are represented in red in Figure 2g,e.

### 3.3. Multilevel Regression Analysis

**Figure 3:**
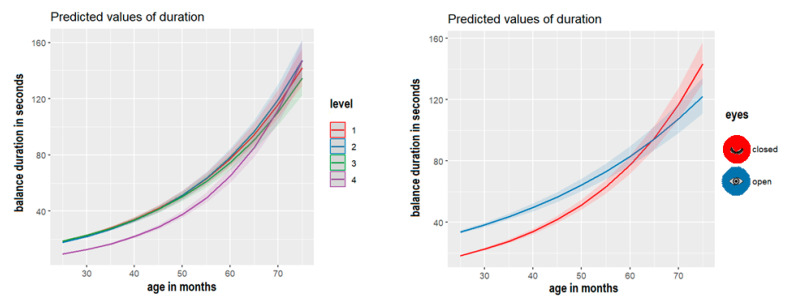
Effect plot estimates and 95% Confidence Interval by M7, showing that the duration of balance stability is predicted by (**left panel**) level of task difficulty across age and (**right panel**) visual modality availability across age.

The model number 7 (M7), including the interaction between age and task difficulty and the interaction between age and visual information availability, emerged to better approximate our data. Supplemental files in the OSF repository show the comparison among the hierarchical models, and these examine the impact of three factors (i.e., age, task difficulty, and visual information availability) on balance duration and balance accuracy. Table 1 and Figure 3 show the estimated effect of M7. Specifically, the most difficult postural tasks—in other words, those that required the movement of the arms (posture 4) and those that involved a lack of visual information availability (closed eyes)—were associated with a reduced duration of postural stability. Note that, as shown in Figure 3, these effects disappear for children older than 5 years of age (60 months), meaning that both the task difficulty effect and the visual information availability effect are modulated by age.

**Figure 4:**
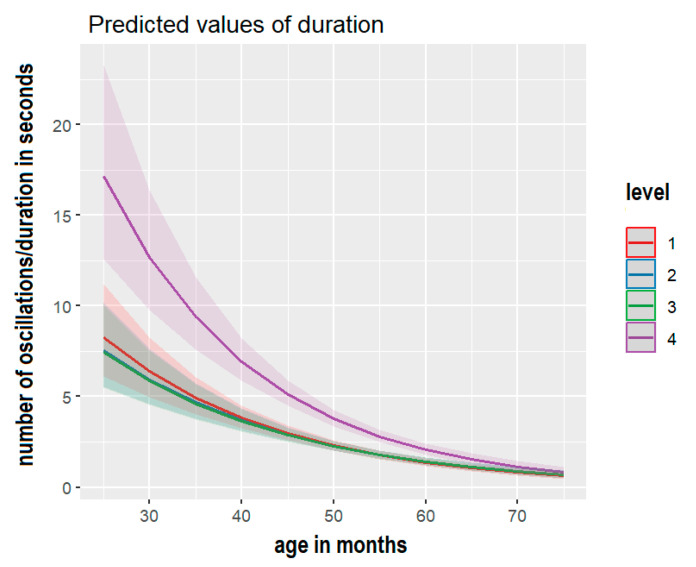
Effect plot estimates and 95% Confidence Interval by M6, showing that balance accuracy is predicted by task difficulty across age in months.

Similarly, model number 6 (M6), including the interaction between age and task difficulty and the fixed factor of visual information availability, was the best approximation of our data. Table 2 and Figure 4 show the estimated effect of M6. Again, the most difficult postural tasks, those that required the movement of the arms (posture 4) in other words, reduced the accuracy of the postural stability, although this effect progressively reduced after 60 months of age, as shown in Figure 4.

Furthermore, the results did not reveal any effect of the lack of visual information (i.e., closed eyes) on the accuracy of postural stability. Lastly, the estimated effects for lexical performance indicated that both lexical comprehension and production substantially increased with age.

### 3.4. Inter-Domain Network Analysis

**Figure 5:**
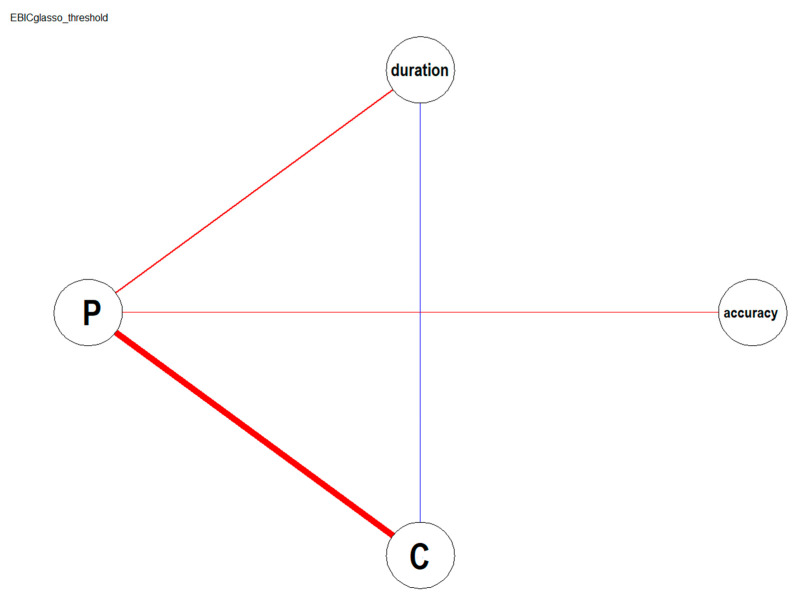
The estimated network represents the partial correlation matrix between C = Comprehension score, P = Production score, duration = Balance duration, and balance accuracy. Positive associations are depicted with red lines, whereas negative associations are depicted with blue lines. The size and color density of the lines (edges) vary to reflect the varying strength of the relationship between the variables; the edges are non-directional, as the data are represented as bivariate partial correlations between the variables.

Figure 5 shows the estimated network of weighted relationships among Language (Comprehension and Production) and Postural (duration and accuracy) measurements. Lexical production is strongly, positively correlated with lexical comprehension and with both the duration and the accuracy measured in the postural tasks. The findings also reveal an interaction between lexical production, comprehension, and the duration of postural stability. Note that lexical production is the node with the highest number of associations with other nodes, suggesting the centrality of the lexical production node within the structure of relationships between the language and postural domains assessed in this study.

**Figure 6:**
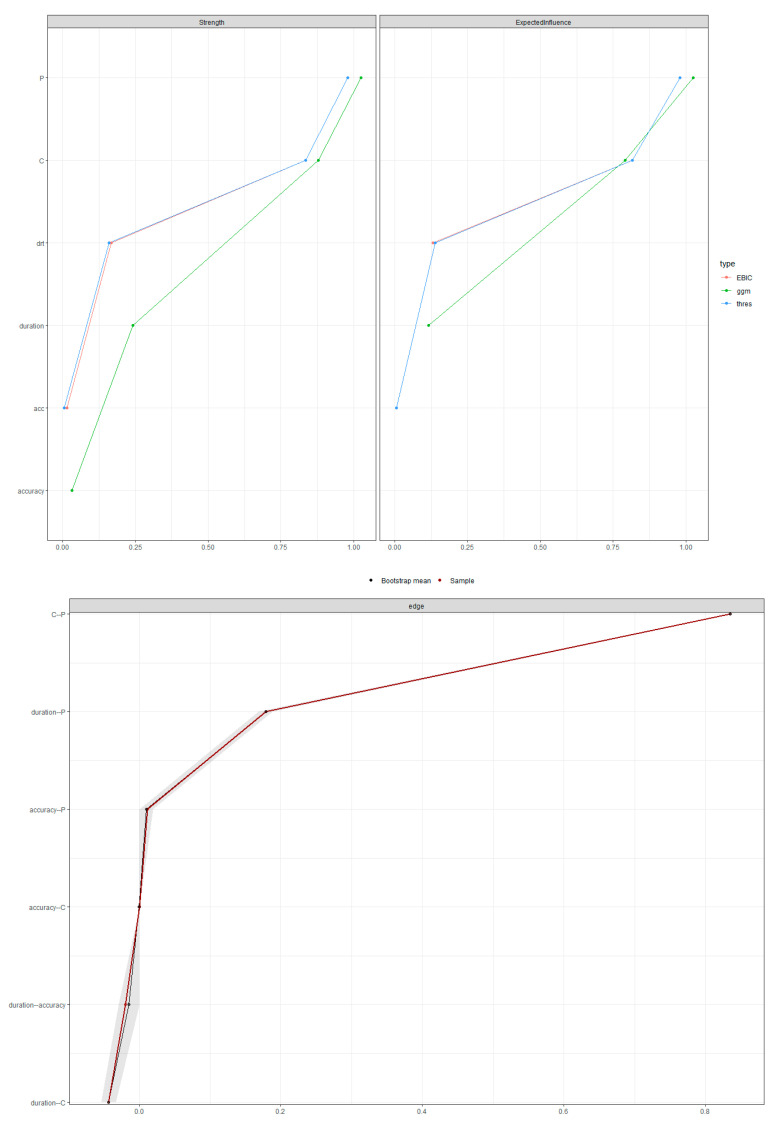
(**upper panel**) Comparison between three networks: EBIC = standard EBISglasso; thres = a more conservative EBICglasso with a threshold, and ggm = unreguralized estimation; (**lower panel**) The *y*-axis provides all the pairwise edges in the network. The red dots provide the edge weights of the network, and the grey area represents the 95% CI around the weights. In both graphs, the *x*-axis indicates the estimated strength for each node.

Figure 6 in the upper panel shows that the conservative GLASSO and the unregularized estimation similarly indicate the centrality of node P (production) within the network compared to all other nodes. Also, to better interpret the network analysis results, the stability of the edge-weight parameters and the edge-weight accuracy was estimated. As shown by Figure 6 in the lower panel, the small gray area indicates that the network analysis offered a stable estimation. Furthermore, checking the stability of the network further confirmed the results, suggesting the central role of lexical production in driving the relationships between postural and language skills.

## 4. Discussion

This study investigated the correlation between lexical skills and postural stability in preschoolers aged 2 to 5 years. It aimed to assess lexical production and comprehension as well as postural stability in terms of duration and accuracy in order to explore the plausible network of associations between linguistic and postural development in early childhood.

First, as a sanity and informative preliminary check, data visualization and missing data inspection revealed that, in lexical tasks, younger children (<50 months of age) showed a higher frequency of missing data in language production compared with comprehension tasks. This pattern descriptively indicated that productive vocabulary skills were still under refinement in younger preschoolers. Moreover, for the postural tasks, the visualization showed a higher incidence of missing data compared to the lexical tasks as well as a consistent distribution of missing data independent from age. Notably, missing data distribution showed the substantial impact of the experimental manipulations, with a substantial amount of missing data in the more complex postural tasks that included arm movement and in the more complex postural tasks when visual information was reduced, such as during closed-eye conditions. Also, these data indicate that the postural task we designed is suitable for measuring postural skills in the preschool period.

Second, the analysis of the inter-domain dataset continued by focusing on each domain individually, applying generalized linear regression analysis in line with explicit confirmatory hypotheses on the data observed. The findings indicated that task difficulty (open arms) and the availability of visual information (closed eyes) significantly impacted postural stability duration by reducing it. This aligns with previous studies showing a decrease in postural stability with variations in body segment alignment, such as in the arms [57], and in conditions of reduced or absent visual cues [42]. Moreover, lexical comprehension and production significantly increased with age. These convergent findings from multiple data (and missing data) underscore the robustness of the lexical and postural stability tasks administered in this study. In particular, the two postural tasks provided distinct and complementary measures of stability, enabling the modeling of individual differences influenced by age, task difficulty, and visual information.

Furthermore, inter-domain network analysis explored the ongoing relationship between postural and language skills in preschoolers [14,15]. This study revealed a strong intra-domain relationship between lexical production and comprehension and a positive inter-domain relationship between lexical production and balance duration. Lexical production emerged as a pivotal node in this inter-domain relationship, linking linguistic proficiency to postural stability. This suggests a reciprocal influence, where children proficient in naming figures also exhibited better postural stability and vice versa. The central role of the lexical production node can be understood by considering the complexity of speech production, which involves coordinating multiple systems and transforming an abstract linguistic message into a motor code [58].

Unlike comprehension, production is rooted in oral motor functions [16]. Post age three, vocabulary production becomes a powerful tool for manipulating the environment, exemplified by directing attention through language [12,59,60]. This interpretation of our findings is rooted in the fact that playing is a child’s main activity at preschool age. To be a proficient player, the child must perform complex motor activities (e.g., jumping, chasing, climbing,) which require speed, coordination, equilibrium, and dexterity, and this improves their ability to comprehend and produce new words and new meanings, especially regarding the names of objects and the verbs related to actions and play, e.g., the fact a ball is an object that can be kicked. After the second year of life, playing begins to be collective through the involvement of other peers, who are less able to tackle their lack of proficiency, inducing an improvement in children’s linguistic abilities in terms of comprehension and production [26]. In other words, the context of play allows for a privileged occasion for children in the building of fruitful relationships with peers by moving and speaking with them. In fact, children with motor and postural difficulties tend to avoid collective playing, preferring solitary play, which reduces their opportunity to develop typical communication and social skills [61]. The present findings extend previous research by Wang et al. (2014) [14,17], indicating the predictive power of language performance on motor skills in children aged 3 to 5. Network analysis provided nuanced insights into the language–motor association in preschoolers, identifying language production efficiency as a key factor in understanding this relationship. This study’s open data and methodological suggestions aim to inform future research that traces the interplay between motor and linguistic domains.

Beyond these strengths, our study has some limitations. First, performance rather than abilities was investigated, as this study only included measurements in a single day per child. Therefore, in addition to our postural tasks, future research could benefit from a closer assessment of the individual differences in postural stability during free and structured play. Accordingly, adopting a longitudinal intensive data collection approach, rather than relying solely on single-occasion measurements, can optimize the informativity of precise indicators of inter-domain relationships across development.

## 5. Conclusions

Language refinement occurs between ages 2 and 5 and promotes successful interactions with the environment and with others. Also, the refinement of postural skills during preschool years is critical, since it supports children in learning new motor skills and aids in their successful response to the increasing demands of the environment. Following this reasoning, our study enriches the existing literature, exploring the network of inter-domain relationships as informative predictors of child development [62,63] by showing that postural and language skills are closely interdependent in preschool age—a period rich with new motor, cognitive, and social experiences for children. In particular, our findings stressed the importance of lexical production, a component of language that involves motor and speech acts, as the bridge between the motor and language domains in preschoolers. By adopting a multimethod approach, such as the one presented here, further research should expand the network to explore potential cultural and contextual factors that may influence language and postural development in preschoolers.

## Figures and Tables

**Table 1:** Estimated linear regression coefficients, standard error (Std. Error), and *p*-values of the selected model (M7) for each factor, i.e., difficulty level (1 as reference level to 2, 3 and 4 in brackets), open vs. closed eyes, and * = the interaction with age, predicting balance duration.

Predictors	Estimates	Std. Error	*p*
(Intercept)	1.90	0.001	<0.01
age	0.04	0.002	<0.01
duration level (2)	−0.08	0.001	<0.01
duration level (3)	0.04	0.001	<0.01
duration level (4)	−0.99	0.001	<0.01
duration eye (open)	0.99	0.001	<0.01
age * duration level (2)	0.002	0.0001	<0.01
age * duration level (3)	−0.001	0.0001	<0.01
age * duration level (4)	0.01	0.0002	<0.01
age * duration eye (open)	−0.015	0.0001	<0.01
Random Effects			
σ^2^	0.30		
τ_00 id_	0.16		
τ_11_ id.duration_shapeU	0.06		
ρ_01 id_	−0.55		
ICC	0.35		
N _id_	173		
Observations	40,224		
Marginal R^2^/Conditional R^2^	0.266/0.525		

**Table 2:** Estimated linear regression coefficients, standard error (Std. Error), and *p*-values of the selected model (M6) for each factor, i.e., difficulty level (from 1 as reference level to 2, 3 and 4 in brackets) and * = the interaction with age, predicting balance accuracy.

Predictors	Estimates	Std. Error	*p*
(Intercept)	3.398	0. 285	<0.001
age	−5.138	0.547	<0.001
accuracy level (2)	−1.82	0.649	0.005
accuracy level (3)	−2.00	0.649	0.002
accuracy level (4)	9.502	0.748	<0.001
accuracy eye (open)	0.002	5.07	1.000
age * accuracy level (2)	3.57	1.21	0.003
age * accuracy level (3)	3.73	1.21	0.002
age * accuracy level (4)	−8.88	1.36	<0.001
age * accuracy eye (open)	−0.005	9.48	1.000
Random Effects			
σ^2^	1.47		
τ_00 id_	0.85		
ICC	0.37		
N _id_	171		
Observations	38,764		
Marginal R^2^/Conditional R^2^	0.115/0.440		

## Data Availability

The data presented in this study are available in Supplementary Materials.

## Supplementary Materials

The following supporting information can be downloaded at: Open Science Framework (OSF) repository https://osf.io/r5sk6/?view_only=66b62d9cda284e9c8584a79c5cc46a1f (accessed on 2 May 2023).
